# Influence of ethnic origin on the clinical characteristics and intestinal flora of irritable bowel syndrome: a prospective study between Han and Tibetan patients

**DOI:** 10.3389/fmed.2024.1359962

**Published:** 2024-04-04

**Authors:** Xiao Ma, Hui Huan, Chao Liu, Hong Hu, Tao Ren

**Affiliations:** ^1^Department of Pediatrics Gastroenterology, West China Second Hospital, Sichuan University, Chengdu, China; ^2^Key Laboratory of Birth Defects and Related Diseases of Women and Children (Sichuan University), Ministry of Education, Chengdu, China; ^3^Department of Gastroenterology, Hospital of Chengdu Office of People’s Government of Tibetan Autonomous Region, Chengdu, China

**Keywords:** irritable bowel syndrome, ethnic origin, gut microbiota, clinical characteristics, 16sRNA Illumina sequencing

## Abstract

**Background:**

Few studies have focused on the clinical characteristics and intestinal flora of Tibetan patients with irritable bowel syndrome (IBS). The study aimed to compare the difference of between Tibetan and Han patients with IBS.

**Methods:**

Patients who met inclusion and exclusion criteria were divided into the Tibet and Han groups. A simplified Gastrointestinal Symptom Rating Scale (GSRS)-based questionnaire was used to assess the IBS severity. Fecal samples from all subjects were collected for the analysis of gut microbiota using 16sRNA Illumina sequencing.

**Results:**

No significant difference was found in the total symptom scores between two groups. However, Tibetans with IBS are more prone to bloating than Hans (17.41% vs 9.09%, *p* < 0.001). A profit shift in the gut microbiota was shown between the two groups. The ratio of Firmicutes/Bacteroidetes was significantly lower in the Tibet group than in the Han group (2.954 ± 0.78 vs 8.23 ± 2.04, *p* = 0.004). In the Tibet group, the level of the genus Blautia decreased significantly compared to the Han group, and there was a significant negative correlation between the level of Blautia and the bloating scores (*Pearson r* = −0.33, *p* = 0.025).

**Conclusion:**

The characteristics of Tibetan patients differ from those of Han patients with IBS, not only in terms of the clinical symptoms, but also in the characteristics of intestinal flora. Tibetans with IBS are more prone to bloating, which might be due to the different gut microbiota. The genus Blautia may play a role in this mechanism.

## Introduction

Irritable bowel syndrome (IBS) is a common functional gastrointestinal disease, characterized by abdominal pain, bloating, and defecation and stool disorders. The symptoms range from diarrhea to constipation, or both, with abdominal pain or discomfort existing alongside abdominal distension (1). The gut microbiome plays a role in symptom development in some patients with IBS (2). The shift in microbiota enterotypes may be an important prognostic factor (3).

Changes in gut microbiota may play an important role in IBS occurrence and development (4). Large differences in the microbiota composition exist among individuals, with differences attributed to age, ethnicity, lifestyle, and diet (5, 6). A number of researchers suggested that ethnic origin contributes to the shaping of the human gut microbial community (7–9). Native Tibetans have evolved to adapt to extreme environments characterized by high radiation, low oxygen levels, and air pressure (10, 11). In addition, the intestinal microflora could be greatly influenced by unique Tibetan food sources, including beef, yak oil, mutton, and a small proportion of vegetables and fruits (12). Their unique living environment, genetic susceptibility and special eating habits make their intestinal flora different from that of the Han population (12).

To the best of our knowledge, few studies have focused on the clinical features and intestinal flora of IBS in the Tibetan population. In this study, we aimed to compare the difference between Tibetan and Han patients with IBS.

## Materials and methods

### Ethnic

This prospective single-center trial was designed and conducted in the Department of Gastroenterology, Hospital of Chengdu Office of People’s Government of Tibetan Autonomous Region, PR China. The protocol was approved by the Medical Ethics Committee of Hospital of Chengdu Office of People’s Government of Tibetan Autonomous Region in Chengdu, China [approval number: (2018) study No. 20]. Written informed consent was obtained from each subject prior to enrollment.

### Subjects

Patients diagnosed with IBS in the outpatient department were included in this study. The inclusion criteria were as follows: IBS were diagnosed according to the Rome IV criteria (13) and aged between 18 and 70 years. The exclusion criteria were a history of gastrointestinal surgery, inflammatory bowel disease, celiac disease, systemic diseases including diabetes, liver cirrhosis, cardiac dysfunction, chronic obstructive pulmonary disease and chronic renal disease; Drug addiction or mental illness; Pregnant or lactating women; treatment with antibiotics, acid suppressants, gastrointestinal stimulants, antidepressants, and opioids 2 weeks prior to study. Subjects who met the inclusion and exclusion criteria were divided into the Tibet group and Han groups according to race.

### Enrollment and sample collection

All the enrolled patients underwent routine treatment. The clinical data of all subjects were recorded. Besides, a simplified GSRS-based questionnaire (14) comprising five dimensions of IBS-related symptoms was designed to assess the severity of IBS. The GSRS questionnaire is a well-validated questionnaire scale designed in 1988 to assess the severity of gastrointestinal symptoms (15). The questionnaire focused on the pain-related symptoms and defecation symptoms, including the severity of abdominal pain and bloating, frequency of abdominal pain and pain during defecation, diarrhea and constipation (Supplementary File 1). In addition, fecal samples of all subjects were collected for analysis of gut microbiota using 16sRNA Illumina sequencing analysis.

### Microbiota analysis

At least 6g of fecal samples were collected and quickly stored at −80°C for further processing. Total genomic DNA was extracted from frozen fecal samples using a DNeasy PowerSoil kit (Tiangen Biotechnology Co., Ltd.) following the manufacturer’s instructions. The quality and quantity of DNA were verified by agarose gel electrophoresis and spectrophotometry. Extracted DNA was diluted to a concentration of 1 ng and stored at −20°C until further processing. Total bacterial genomic DNA was extracted and the V3 variable regions of the 16S rRNA genes were amplified by PCR using the universal primers 338F_806R. 16S rRNA gene analysis of fecal samples was performed as described previously (16). An Illumina sequencing adaptor was added to the primers, and the reverse primer contained a sample barcode. The quality of the amplicons was observed using gel electrophoresis; the PCR products were then purified. The concentration was adjusted using an Illumina MiSeq PE 300 system (China Shanghai Maibo Bio-pharmaceutical Technology Co., Ltd.) for sequencing. The 16S rRNA gene data were processed on the Majorbio Cloud Platform.^1^ After filtering the sequencing reads and constructing feature table, operational taxonomic units (OTUs) were picked at 97% identity cut-off. All data were analyzed at the domain-to-species level. The Wilcoxon rank-sum test was used to detect the different levels of differential abundance. Chao1 and Simpson’s indices were used to visualize α diversity, while principal coordinate analysis was used to visualize the β-diversity, showing the richness and diversity of the community. The linear discriminant analysis effect size (LEfSe) and Linear discriminant analysis (LDA) was used to find unique bacterial taxa among different groups (17). Diversity and richness plots were generated in GraphPad Prism (version 6.0). Beta-diversity was measured by calculating phylogenetically based Bray-Curtis index. Principal coordinate analysis (PCoA) was applied to the resulting distance matrices to generate plots using the default settings of PRIMER-635.

### Statistical analysis

GraphPad Prism (version 6.0) and SPSS software version 20.0 (SPSS Inc., Armonk, New York, USA) were adopted for statistical analysis. Between-group differences were evaluated using Student’s t-test for continuous variables and Pearson’s χ^2^ test for categorical variables. Kruskal–Wallis or Mann–Whitney test was used to compare the OTUs and taxonomy abundances. The resultant *p*-values were FDR (false discovery rate) with a significance threshold of 5%. Pearson’s correlation analysis was used to analyze the correlation between IBS symptoms and specific flora. Two-tailed *P*-values were generated for all parameters, and *P*-values < 0.05 were considered significant. Other statistical analyses, including Wilcoxon rank-sum Test, were performed.

## Results

### Patient characteristics

From August 2018 to January 2019, a total of 45 patients with IBS were included, including 20 Han patients and 25 Tibetan patients, aged from 27 to 74. All the participants signed an informed consent form and volunteered to participate in this study. The baseline data for the two groups were comparable (Table 1). No significant difference was found in the total scores of symptom between the Han and Tibetan patients from the symptoms questionnaire.

**TABLE 1:** Baseline characteristics.

	Tibet	Han	*P-*value
	(*n* = 25)	(*n* = 20)	
Age (mean, SD)	47.84 (2.05)	52 (2.04)	0.16
Gender M/F	16/9	12/8	0.78
Body mass index(kg/m^2^)	25.44 (0.70)	23.93 (0.82)	0.16
(Mean, SD)			
Duration of IBS symptoms			0.88
3 months to 1 years	8	6	
>1 years	17	14	
Total score of questionnaire (mean, SD)	7.04 (2.263)	6.16 (2.19)	0.12

SD, standard deviation.

### Tibetans with IBS are more prone to bloating

**FIGURE 1:**
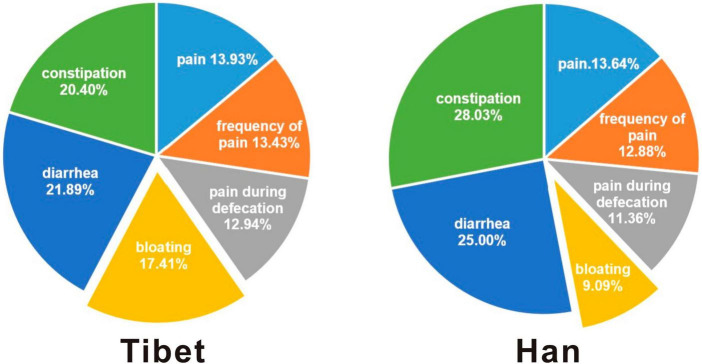
Five-dimensions for the GSRS based questionnaire in two groups.

Although there was no significant difference in the total of IBS symptom scores between the two groups, the symptoms are differed in each dimension (Table 2). According to the symptom questionnaire (Figure 1), there was no significant difference between the Tibetan and Han patients in pain-related symptoms such as abdominal pain (13.93% vs 13.64%), pain frequency (13.43% vs 12.88%) and defecation pain (12.94% vs 11.36%). The defecation symptoms, such as diarrhea (21.89% vs 25%) and constipation (20.4% vs 28.03%), of the Han and Tibetan patients were similar. However, it should be noted that Tibetans with IBS are more prone to bloating, and the proportion is significantly higher than that of the Han people (17.41% vs 9.09%, *p* < 0.001).

**TABLE 2:** Mean (SD) scores for GSRS-based questionnaire in two groups.

	Tibet	Han	*P-*value
	(*n* = 25)	(*n* = 20)	
Total score	7.04 (2.263)	6.16 (2.19)	0.12
Abdominal pain	0.98 (0.6)	0.84 (0.53)	0.42
Frequency of pain	0.95 (0.43)	0.79 (0.6)	0.3
Pain during defecation	0.91 (0.93)	0.7 (0.88)	0.45
Bloating	1.23 (0.71)	0.56 (0.56)	<0.01
Diarrhea	1.51 (0.55)	1.54 (1.07)	0.9
Constipation	1.44 (0.66)	1.73 (0.95)	0.23

SD, standard deviation.

### Characteristics of the gut microbiota in Tibetans and Hans with IBS

All patients were included in the microbial analysis (25 cases in Tibet group and 20 cases in Han group). Illumina paired-end sequencing of fecal mucosa generated an average of 52900 high-quality 16S rRNA gene sequences (range: 30562–73424) and 823 OTUs. At an even length of 10000 sequences per sample, OTUs at a 97% similarity threshold for microbiota were clustered.

**FIGURE 2:**
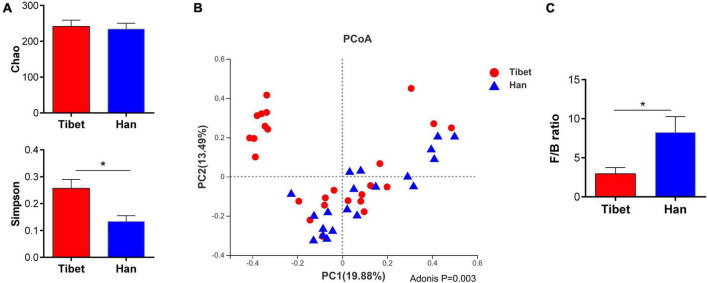
Comparison of α and β diversity between two groups. **(A)** α diversity indexes between two groups (ANOVA analysis); **(B)** Principal coordinate analysis (Bray-Curtis index) of all samples at the OUT level revealed distinct clustering of fecal microbiota between two groups, *P* = 0.003. **(C)** Firmicutes to Bacteroidetes (F/B) ratio in Tibet and Han patients with IBS.

**FIGURE 3:**
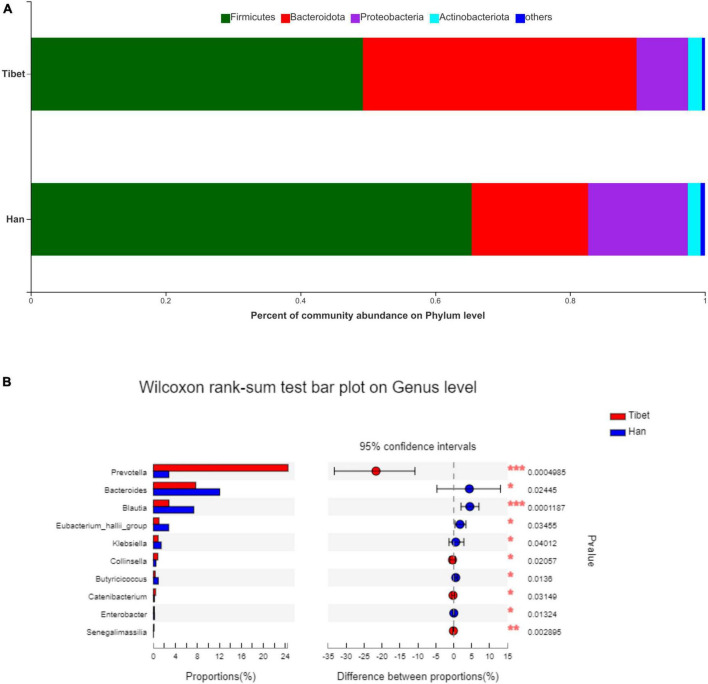
Taxonomic composition of samples at phylum and genus level in two groups. **(A)** Taxonomic composition of samples at phylum level. **(B)** Taxonomic composition of samples at genus level.

No significant difference was found between the Tibetan and the Han groups in the Chao indices of α-diversity, but Simpson diversity was significantly decreased (Figure 2A). Principal co-ordinate analysis (Bray-Curtis index) of all samples at the OTU level revealed clustering of gut microbiota in Tibet patients that was distinct from the Han group, *p* = 0.003 (Figure 2B). The heatmap further demonstrates the difference in gut microbiota between Tibetan and Han populations (Supplementary Figure 1). At the phylum level, the proportion of Bacteroides in Tibet group was significantly higher than that in Han group (38.16 ± 28.15% vs 17.28 ± 13.72%, *p* = 0.015; Figure 3A). The Firmicutes/Bacteroidetes (F/B) ratio was significantly lower in the Tibet group than the Han group (2.954 ± 0.78 vs 8.23 ± 2.04, *p* = 0.004 by Mann–Whitney U test; Figure 2C). In total, 20 taxa were significantly different between the Tibet and the Han group and the top-10 representative taxa were showed in the Figure 3B. At the genus level, the enrichment of Prevotella, Collinsella, Senegalimassilia and Catenibacterium was found in the Tibet group, whereas the relative abundance of the genera Blautia, Bacteroides, Eubacterium_hallii, Klebsiella, Butyricicoccus and Enterobacter decreased compared to that in the Han group.

### Bloating might link to the characteristics of gut microbiota

**FIGURE 4:**
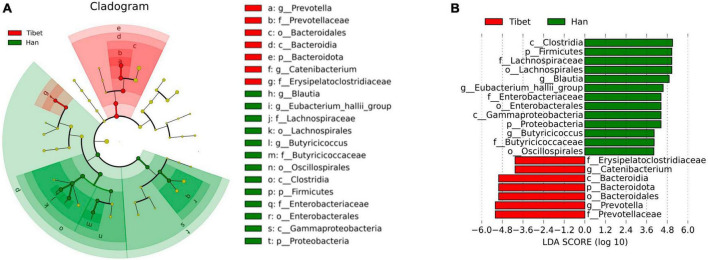
LEfSe **(A)** and LDA **(B)** analysis of microbiota changes of two groups.

Bacterial overexpression at different taxonomic levels due to race populations was characterized using LEfSe and LDA methods (Figure 4). There were profound changes in the gut microbiota in both groups. Compared to the Han group, the genus Blautia, belonging to the phylum Firmicutes in the Tibet group significantly decreased, while the genus Prevotella, which belonged to the phylum Bacteroidetes, was enriched. We analyzed the correlation between the bloating score and the potential predictive bacterial genera Blautia and Prevotella. There was a significant negative correlation between the genus Blautia and the bloating scores (*Pearson r* = −0.33, *p* = 0.025).

## Discussion

The etiology of IBS is not fully understood, however, several factors such as genetics, diet, and gut microbiota are thought to play a role in IBS pathophysiology (2). Genetic, environmental and dietary habit have been reported to affect the gut microbiota. In this study, the characteristics of the appearance and gut microbiota of the Tibetan population with IBS was firstly are reported for the first time. The Tibetans were more prone to bloating than the Han population, which might be due to the shift in the gut microbiota.

In this study, we found that Tibetan patients with IBS also exhibited different symptom characteristics, such as abdominal pain and abnormal bowel movements, but were more prone to abdominal distention than Han patients. The unique gut microbiota structure has been reported to be related to the severity of the psychological and gastrointestinal symptoms of IBS (3). Furthermore, diet plays an important role in the pathophysiology of IBS and appears to result from the interactions between intestinal bacteria and endocrine cells (18). Therefore, the specific dietary structure of Tibetans may shape their unique intestinal environment and flora, thus affecting the abundance of gas-producing bacteria and making Tibetans more susceptible to bloating.

Therefore, this study compared the differences in gut microbiota between Tibetan and Han patients with IBS using 16S rDNA high-throughput sequencing. Profound shifts have been observed changes were found in the gut microbiota of between the Hans and the Tibetans patients with IBS. In the present study, the species diversity of the gut microbiota, as represented by the Simpson index was significantly higher in Tibetans than that in Hans with IBS. The F/B ratio, which is regarded as having an important influence on maintaining normal intestinal homeostasis (19), is significantly lower in Tibetans. Some studies have reported that the b/f ratio in patients with IBS was lower than that of healthy individuals (20). These results indicate that the intestinal flora of Tibetan patients with IBS is more dysbiosis than that of the Han individuals. At the genus level, the enrichment of *P*revotella, *C*ollinsella, *S*enegalimassilia and *C*atenibacterium was found in the Tibet group, whereas the relative abundance of genus *B*lautia, *B*acteroides, *E*ubacterium_hallii, *K*lebsiella, *B*utyricicoccus and *E*nterobacter were decreased compared to the Han group. The over-representation of bacteria at different taxonomic levels in response to race origin as characterized using LEfSe and LDA methods. According to the LDA analysis, the genus *Blautia* belonging to the phylum Firmicutes was significantly enriched in the Han patients, while the genus *Prevotella*, which belonged to the phylum Bacteroidetes, was enriched in the Tibet group. It has been demonstrated that genus *P*revotella can exert its proinflammatory effect that leads to increased intestinal permeability and mucosal inflammation (21–23). The genus *Blautia* is a butyrate-producing bacterium with anti-inflammatory properties, which may reflect gas levels in the intestinal tract by converting intestinal gas into short-chain fatty acids (24, 25). In this study, the species levels of Blautia were observed to decrease among the Tibetan people. In addition, the decline at the species level in *Blautia* was negatively correlated with the bloating symptom score in this study, which should be addressed.

This study has some limitations. This was a single-center study, which may have biased the data. A well- designed, multiple-center study would be more convincing. A limited number of IBS patients with IBS were included in this study, and healthy controls were could be further enrolled to make this study more rigorous. More samples and further molecular biology experiments are required to verify the results of this study, and the classification of IBS should be discussed in more detail.

In summary, Han and Tibetan patients with IBS exhibited unique characteristics, both in terms of symptoms and differences in gut microbiota. Tibetans with IBS are more prone to bloating, which may be due to the gut microbiota. Thus, the genus *Blautia* may play a role in this mechanism.

## Data availability statement

All data generated or analyzed during this study are included in this published article (and its Supplementary material).

## Ethics statement

The studies involving humans were approved by the Medical Ethics Committee of Hospital of Chengdu Office of People’s Government of Tibetan Autonomous Region in Chengdu, China. The studies were conducted in accordance with the local legislation and institutional requirements. The participants provided their written informed consent to participate in this study.

## Author contributions

XM: Writing – review and editing, Writing – original draft, Visualization, Software, Methodology, Formal analysis, Data curation. HuH: Writing – review and editing, Supervision, Project administration, Investigation, Funding acquisition. CL: Writing – review and editing, Supervision, Software, Resources, Project administration, Investigation. HoH: Writing – review and editing, Methodology, Investigation, Formal analysis, Data curation. TR: Writing – review and editing, Visualization, Validation, Software, Resources, Formal analysis.

## Footnotes

1
www.majorbio.com

## Supplementary material

Supplementary Figure 1 The heatmap of gut microbiota between Tibetan and Han.
