# Supplementary figures and images for: Influence of ethnic origin on the clinical characteristics and intestinal flora of irritable bowel syndrome: a prospective study between Han and Tibetan patients
Source: Front Med (Lausanne). 2024 Apr 4;11:1359962. doi: 10.3389/fmed.2024.1359962 (PMC11024223; doi:10.3389/fmed.2024.1359962)

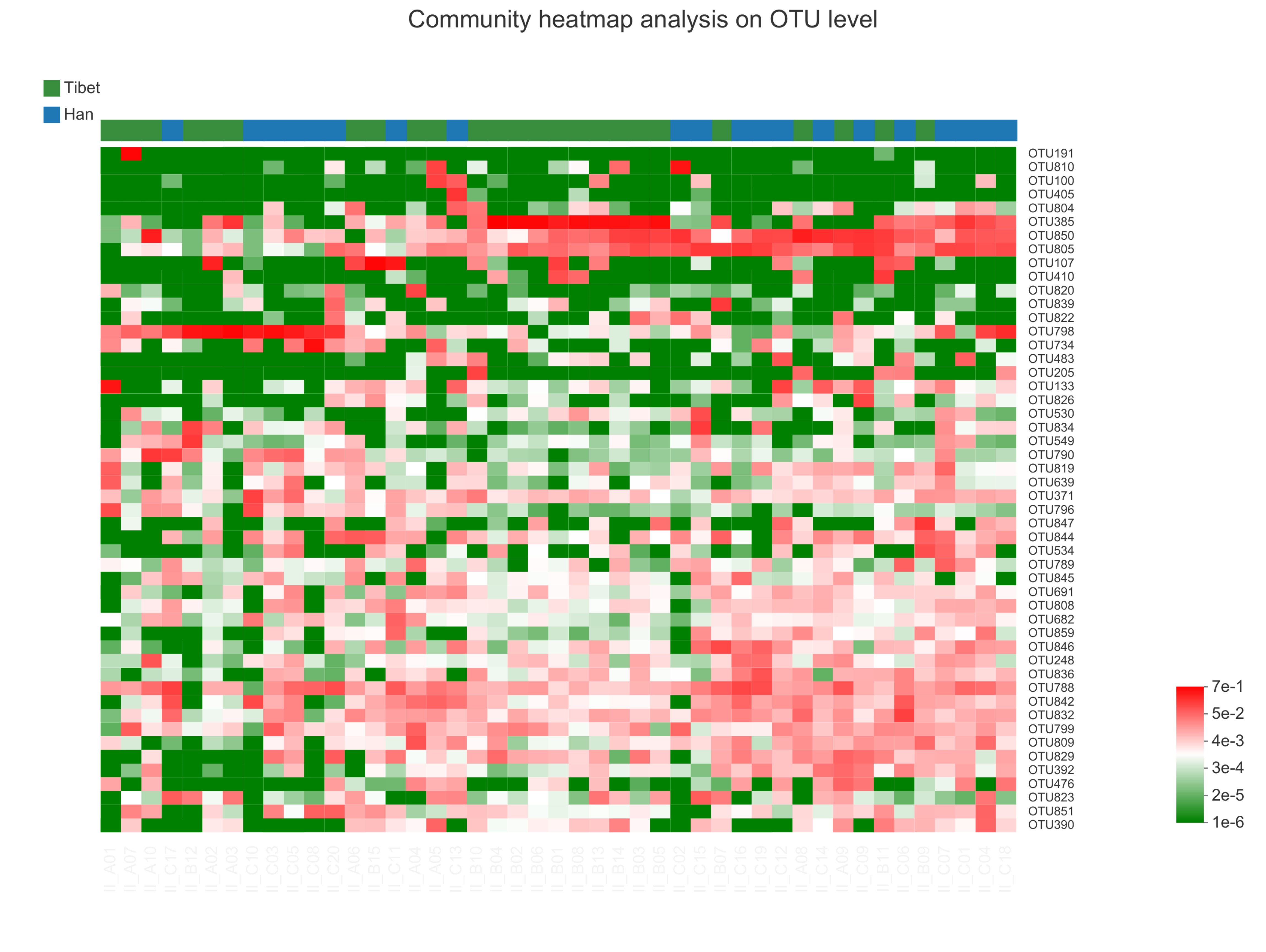

Supplement: Supplementary Figure 1 — The heatmap of gut microbiota between Tibetan and Han. [file Image_1.JPEG]
