# Supplementary material for: Influence of ethnic origin on the clinical characteristics and intestinal flora of irritable bowel syndrome: a prospective study between Han and Tibetan patients
Source: Front Med (Lausanne). 2024 Apr 4;11:1359962. doi: 10.3389/fmed.2024.1359962 (PMC11024223; doi:10.3389/fmed.2024.1359962)
Supplement: Supplementary file 2 [file Data_Sheet_1.docx]

**GSRS-based questionnaire**

Name: Gender: Age: ID: Total score:

Hello, please fill out the following form truthfully based on your average episodes of disease-related symptoms over the past two weeks, thank you for your cooperation.

| **Symptoms** | **0** | **1** | **2** | **3** | **Score** |
| --- | --- | --- | --- | --- | --- |
| Abdominal pain | No | Minor | Moderate | Severe |  |
| Bloating | No | Minor | Moderate | Severe |  |
| Abdominal pain (hours/day) | 0 | 2 | 2-7 | >7 |  |
| Pain during defecation (%) | 0 | 0-40 | 40-60 | >60 |  |
| Diarrhea (days/week) | 0 | 1-2 | 3-5 | 6-7 |  |
| Constipation  (days/week) | 无 | 1-2 | 3-5 | 6-7 |  |
